# Chemical formation of hybrid di-nitrogen calls fungal codenitrification into question

**DOI:** 10.1038/srep39077

**Published:** 2016-12-15

**Authors:** Rebecca L. Phillips, Bongkeun Song, Andrew M. S. McMillan, Gwen Grelet, Bevan S. Weir, Thilak Palmada, Craig Tobias

**Affiliations:** 1 Landcare Research, Gerald Street, Lincoln, New Zealand; 2 Dept. of Biological Sciences, Virginia Institute of Marine Science, Gloucester Point, Virginia, USA; 3 Dept. of Marine Sciences, University of Connecticut, Groton, Connecticut, USA

## Abstract

Removal of excess nitrogen (N) can best be achieved through denitrification processes that transform N in water and terrestrial ecosystems to di-nitrogen (N_2_) gas. The greenhouse gas nitrous oxide (N_2_O) is considered an intermediate or end-product in denitrification pathways. Both abiotic and biotic denitrification processes use a single N source to form N_2_O. However, N_2_ can be formed from two distinct N sources (known as hybrid N_2_) through biologically mediated processes of anammox and codenitrification. We questioned if hybrid N_2_ produced during fungal incubation at neutral pH could be attributed to abiotic nitrosation and if N_2_O was consumed during N_2_ formation. Experiments with gas chromatography indicated N_2_ was formed in the presence of live and dead fungi and in the absence of fungi, while N_2_O steadily increased. We used isotope pairing techniques and confirmed abiotic production of hybrid N_2_ under both anoxic and 20% O_2_ atmosphere conditions. Our findings question the assumptions that (1) N_2_O is an intermediate required for N_2_ formation, (2) production of N_2_ and N_2_O requires anaerobiosis, and (3) hybrid N_2_ is evidence of codenitrification and/or anammox. The N cycle framework should include abiotic production of N_2_.

The nitrogen (N) removal pathway known as denitrification is typically considered a biological process, where nitrate (NO_3_^−^) or nitrite (NO_2_^−^) is sequentially reduced by bacteria and archaea to nitric oxide (NO), nitrous oxide (N_2_O) and finally inert di-nitrogen (N_2_)^1^. Incomplete denitrification results in emission of N_2_O, an important greenhouse gas now playing a primary role in stratospheric ozone depletion^2^. While prokaryotes are well known to denitrify^3^, new findings indicate denitrification by eukaryotes, such as fungi, may be widespread^4^,^5^,^6^. Unlike prokaryotes, fungi do not have the genes encoding nitrous oxide reductase, which reduces N_2_O to N_2_, so fungal denitrification terminates at N_2_O^7^,^8^. Denitrification occurs when a single N source is used to produce N_2_O, such as NO_2_^−^ or NO_3_^−^. Codenitrification occurs when individual atoms of the N_2_O or N_2_ molecules are derived from two distinct N sources^9^,^10^, resulting in hybrid N_2_^8^. Formation of hybrid N_2_ is widely reported as evidence of anammox^11^ or codenitrification^12^,^13^,^14^. Previously, isotope pairing experiments revealed chemodenitrification^15^,^16^ and denitrification by the fungi *Bipolaris sorokiniana* used NO_2_^−^ as the sole source for N_2_O formation^16^. Fungal denitrification rates of NO_2_^−^ to N_2_O were similar under both anaerobic and microaerophilic conditions, contrary to classical denitrification, suggesting O_2_ may not, in this case, be a strong regulator^16^.

Incubation experiments with pure cultures and soils report a number of fungi may play a significant role in soil N trace gas production^5^,^6^,^17^,^18^. Fungi not only denitrify to N_2_O but may also codenitrify to form N_2_^8^,^9^. When O_2_ is not available, some fungi (*Fusarium oxysporum*) reportedly co-metabolise organic forms of N to reduce NO_2_^−^ or NO_3_^−^ and form hybrid N_2_^7^,^9^,^10^. This has been demonstrated in soils by tracing ^29^N_2_ and ^30^N_2_ following application of antibiotics to selectively inhibit bacteria or fungi^12^,^14^. Codenitrification is widely viewed as an anaerobic, enzymatically-mediated nitrosation process requiring low (<−1) formal oxidation state of the nucleophilic N^8^. Figure 1 illustrates how anammox, like codenitrification, also forms hybrid N_2_, although anammox uses two forms of inorganic N, NO_2_^−^ and ammonium (NH_4_^+^)^11^,^19^.

The specific codenitrification pathway is unknown, but fungi reportedly reduce NO_2_^−^ to NO using NO_2_^−^ reductase (encoded by the *nirK* gene) and then reduce NO to N_2_O using nitric oxide reductase (P450nor)^7^. The role of N_2_O in the codenitrification process and in N_2_ formation is not clear^20^. While utilisation of N_2_O to form N_2_ has been suggested as a plausible codenitrification pathway^8^, reports of N_2_O consumption during fungal production of N_2_ (commonly observed during bacterial denitrification)^21^ are lacking. Potentially bypassing reduction of N_2_O to form N_2_, in addition to formation of hybrid N_2_, sets codenitrification and anammox apart from classical denitrification^22^.

Laboratory studies commonly report evidence of fungal denitrification or codenitrification when pure cultures are incubated under anaerobic or microaerophilic conditions with sterile media, consisting of carbon, NO_2_^−^ and mineral salts^5^,^6^,^9^,^10^,^16^. However, reduced metals in the medium, such as Fe(II), could provide electrons required for abiotic reduction of NO_2_^−^ to N_2_O, commonly known as chemodenitrification^15^,^23^,^24^. Chemodenitrification occurs through nitrosylation when reduced forms of inorganic N react with a metal centre to form N_2_O in the absence of oxygen^15^,^24^. Nitrosylation may also drive abiotic formation of N_2_, given high concentrations of metal and NO_2_^−^ 25. Wullstein and Gilmour^25^ mixed 10,000 ppm N as potassium nitrite (KNO_2_) with 5,000 ppm ferrous sulfate in an abiotic, anoxic reactor and recovered 15% of added N as N_2_ within 3 d. In this case, the N_2_ formed would have been denitrified from a single N source, KNO_2_. Alternatively, chemical formation of hybrid N_2_ through nitrosation is often ignored by biologists. Abiotic nitrosation of organic matter by NO_2_^−^ in soil was first suggested by Nelson and Bremner^26^ when they recovered over 20% of added N (5 mmols NO_2_^−^ g^−1^ soil) as N_2_ for sterile soil at neutral pH in helium (He) and heliox (20% O_2_, 80% He) atmospheres. The isotopic composition of N_2_ was not reported, but they indicated soil organic matter was an important factor^26^. Trimmer and Prudy^27^ showed that deep seawater samples amended with ^15^NO_2_^−^ and ^14^NH_4_ produced more ^29^N_2_ when organic N (allylthiourea) was added. They suggested an alternative metabolic pathway to anammox but did not address the possibility of abiotic ^29^N_2_ formation. Babbin *et al*.^28^ also found organic N enhances anammox N_2_ production. A common thread among chemical, anammox and fungal denitrification studies is NO_2_^−^. Thus, NO_2_^−^ is a pivot-point for divergence in biological and chemical N trace gas production^29^ (Fig. 1).

Here, we pursue open questions raised by this early work regarding abiotic N trace gas production recently reviewed by Heil *et al*.^30^. We aimed to investigate if previously unexplored sources of N_2_O and N_2_ could be contributing to reactive N removal and if N_2_O was an intermediate in the abiotic N_2_-production pathway. We questioned whether N_2_ reportedly due to fungal codenitrification in pure culture experiments was formed abiotically in the presence and absence of O_2_, given diverse inorganic and organic sources of N. New knowledge of abiotic N_2_O and N_2_ production would advance environmental N-removal research and applications and perhaps explain some mass balance discrepancies found in isotopic pairing studies^27^,^28^.

To address these questions, we used a ubiquitous soil fungus, *Bipolaris sorokiniana* (Sacc.) Shoemaker [telemorph: *Cochliobolus sativus* (S. Ito & Kurib.) Drechsler ex Dastur], as our model. Previously, we found that *B. sorokiniana* used NO_2_^−^ as the sole source for denitrification to N_2_O under anaerobic and microaerophilic conditions^16^. We also found NO_2_^−^ as the sole source for chemodenitrification, which accounted for 6–8% of total N_2_O production^16^. These results prompted further inquiry regarding fungal and chemical trace gas production of N_2_. We aimed to more explicitly evaluate abiotic and biotic N_2_O and N_2_ production by comparing live fungi with fungal necromass incubated under strictly sterile conditions, with and without O_2_. Pure culture experiments have demonstrated fungal and chemical N_2_O production, but reports are lacking that indicate abiotic nitrosation of organic compounds and formation of hybrid N_2_. We include necromass in the design because amino acids and other nucleophilic compounds from necromass could potentially, in the absence of live fungi, react with NO^2−^ to form N_2_. Further, necromass would provide more surface area for chemical decomposition of NO_2_^−^ to N_2_O^24^ (Fig. 1). This would serve as a test for abiotic formation of N_2_O and N_2_ in the presence of decomposing organic material.

We aimed to assess biotic and abiotic N_2_O and N_2_ production using established microbiological laboratory incubation methods (Fig. 2). We exposed live fungi and necromass to both inorganic and organic N sources [0.25 mmol N as sodium nitrite (NaNO_2_) and 0.25 mmol N as glutamine (C_5_H_10_N_2_O_3_); concentration of 20 mmol L^−1^ each] under two O_2_ conditions (anaerobic and 20% O_2_). For each treatment, there were replicate sets where N was not added to the media (No N control). We also included sterile media-only control vessels. Oxygen status was tightly controlled with airtight laboratory incubation vessels, where headspace was filled with either helium (anaerobic) or heliox (aerobic). Accumulation of headspace O_2_, CO_2_, N_2_O, and N_2_ were measured approximately every 6 h with a customised, robotic gas chromatography system^21^. Headspace O_2_ was monitored to confirmed anaerobiosis was maintained during the 30 h incubation. Slopes of the linear increases in N_2_O and N_2_ in the headspace of each vessel were calculated to determine production rates. We used ANOVAs to test if production of N_2_O and N_2_ by live fungi was similar to necromass, and if both groups responded similarly to O_2_. Production rates were calculated in units of μmol N as N_2_O and N_2_ g fungal biomass^−1^ h^−1^ and in units of μmol N as N_2_O and N_2_ h^−1^. We performed a second incubation (illustration of this incubation design not shown) using isotope pairing techniques to determine if ^29^N_2_O or ^30^N_2_O were produced abiotically from sterile medium amended with glutamine and NO_2_^−^ in the presence or absence of O_2_. We used equal amounts of unlabelled glutamine and ^15^N-labelled NO_2_^−^ to achieve 0.5 mmol N and 1.0 mmol N, as well as a medium that was not amended with N.

## Results and Discussion

We found both fungal states (live fungi and necromass) produced N_2_O and N_2_ but only for those samples amended with N. Linear production of N_2_O and N_2_, following N amendment, occurred quickly under completely anaerobic conditions and in a 20% O_2_ atmosphere (Fig. 3). Average [(standard deviation (SD)] rate of N_2_O produced by necromass was 0.012 (<0.001) μmol N_2_O g biomass^−1^ h^−1^ under both aerobic and anaerobic conditions. Average N_2_O production by live fungi was 0.017 (<0.001) μmol N_2_O g biomass^−1^ h^−1^ and 0.028 (0.003) μmol N_2_O g biomass^−1^ h^−1^, respectively, under aerobic and anaerobic conditions (Fig. 3a). Rates of N_2_O production were significantly greater for live fungi incubated anaerobically (O_2_ × fungal state interaction; p < 0.001), indicating biological production of N_2_O in the absence of O_2_. In a 20% O_2_ atmosphere, rates of N_2_O production were similar to necromass. Our previous study indicated *B. sorokiniana* produced N_2_O at similar rates when incubated anaerobically and in a 0.4% O_2_ atmosphere^16^. Here, we used 20% O_2_, and we found no evidence of microbial denitrification under these high-O_2_ conditions.

Figure 4a provides a basis of comparison for N_2_O recovered in the headspace of sterile media using comparable units (μmol N_2_O h^−1^). These data were not normalised per g of fungal biomass but illustrate accumulation of N_2_O in sterile medium relative to necromass and live fungi. Nitrous oxide production rates were greater for necromass, as compared to media only (Fig. 4a). Necromass produced an average of 0.0034 μmol N_2_O h^−1^ at both O_2_ levels, as compared to 0.0011 μmol N_2_O h^−1^ for sterile media. Greater N_2_O for necromass suggests the presence of decaying biomass provided greater surface area for chemodenitrification^24^ (Fig. 4a).

Rates of fungal N_2_O production observed here are lower than other reports in the literature. Maeda *et al*.^6^ found fungi carrying the *nirK* gene produced from 0.1 to 3.2 μmol N_2_O g biomass^−1^ h^−1^, and Rohe *et al*.^31^ reported fungal N_2_O production rates from 0.05 to 14 μmols N_2_O h^−1^. In both cases, denitrification varied with fungal species and N amendment. Here, *B. sorokiniana* does not carry the *nirK* gene, which may influence NO_2_^−^ reduction to NO. Data on chemical formation of N_2_O under oxic (20% O_2_) conditions are lacking, so these first results challenge the paradigm that chemodenitrification requires anoxia^15^. Contrary to chemostat studies, we included fungal necromass, which contributed to abiotic N_2_O. Results call into question if all N_2_O produced in pure culture experiments^6^,^18^,^31^ is enzymatically mediated if cultures include live and dead fungal or bacterial biomass.

We found no evidence of biological N_2_ production in the presence or absence of necromass (Fig. 3b). Lowest rates of N_2_ production were observed for live fungi incubated in a 20% O_2_ atmosphere (O_2_ × fungal state interaction; p < 0.001). Rates of N_2_ produced in the headspace of live fungi were 0.243 (0.039) μmol N_2_ g biomass^−1^ h^−1^ and 0.424 (0.049) μmol N_2_ g biomass^−1^ h^−1^ under aerobic and anaerobic conditions, respectively. Rates of N_2_ produced in the headspace of necromass were 0.415 (0.041) μmol N_2_ g biomass^−1^ h^−1^ and 0.356 (0.047) μmol N_2_ g biomass^−1^ h^−1^ under aerobic and anaerobic conditions, respectively. Our results indicate there is a strong abiotic component to measured rates of N_2_ production and challenge the assumption that fungal N_2_ production^7^ requires anoxic or microaerophilic conditions^8^.

Most of the N_2_ accumulated in the headspace for live *B. sorokiniana* was commensurately found in the headspace of *B. sorokiniana* necromass (Fig. 3b), which points to chemical N_2_ formation. We show chemical production of N_2_ (μmol h^−1^) for sterile medium relative to necromass and live fungi at both O_2_ levels in Fig. 4b. Overall average rates of N_2_ formation were 0.12 (0.039) μmol N_2_ h^−1^ for sterile medium, 0.11 (0.024) μmol N_2_ h^−1^ for necromass, and 0.08 (0.018) μmol N_2_ h^−1^ for live fungi. Others have reported much higher rates of anoxic chemical N_2_ formation (7–32 μmol N_2_ h^−1^) at neutral pH when high concentrations of reduced metals were reacted with high concentrations of NO_2_^−^25 and when high NO_2_^−^ solutions (20 M) were added to autoclaved soil^26^, but these did not report if hybrid N_2_ was formed or not. Comparable rates of abiotic N_2_ production for necromass and sterile medium caused us to question if abiotic N_2_, like N_2_O, could result from nitrosylation of inorganic N only or from nitrosation to form hybrid N_2_. Organic N has been found to increase biological production of ^29^N_2_ in oceanic studies^32^, but our results (Fig. 4b) indicated there may also be abiotic processes that contribute strongly to ^29^N_2_ production. Consequently, we determined if both inorganic and organic N were used to produce N_2_ abiotically in a separate, isotope pairing experiment.

The headspace above sterile media incubated under oxic and anoxic conditions with ^15^N-labelled NaNO_2_ and unlabelled C_5_H_10_N_2_O_3_ indicated abiotic, formation of hybrid N_2_. Almost all (99.9%) of the N_2_ produced was ^29^N_2_ (Table 1). The ^29^N_2_ production was proportional to the mass of added N and approximately 3–4% of the total N added was transformed to ^29^N_2_ under both oxic and anoxic conditions and at both levels of NO_2_^−^ addition. Results demonstrate hybrid formation of N_2_ is not necessarily enzymatically mediated and does not required anoxia. Formation of abiotic, hybrid N_2_ by two distinct inorganic N molecules has not been ruled out here. Previous bodies of work that use formation of ^29^N_2_ as evidence of anammox and/or codenitrification^12^,^14^,^32^,^33^ need to be reviewed with respect to abiotic N_2_ production.

**Figure 1:**
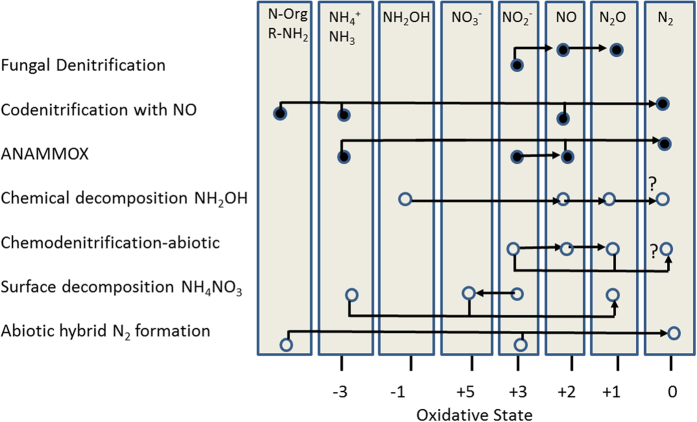
Schematic of codenitrification, anammox and known chemical denitrification pathways. Selected processes potentially leading to N_2_O and N_2_ formation, involved N compounds, their reaction pathways as well as their oxidation states are shown. Closed circles are biotic and open circles are abiotic reactions. Terms are defined as follows: Norg/R-NH_2_, monomeric organically bound N forms; NH_4_^+^, ammonium; NH_3_, ammonia; NH_2_OH, hydroxylamine; NO_2_^−^, nitrite; NO_3_^−^, nitrate; NO, nitric oxide; N_2_O, nitrous oxide; N_2_, molecular dinitrogen. The last process, abiotic N_2_ formation, was observed in this study. Figure 1 is a truncated adaptation from Butterbach-Bahl *et al*.^23^, which is licensed under a Creative Commons Attribution License 3.0 (https://creativecommons.org/licenses/by/3.0/).

**Figure 3:**
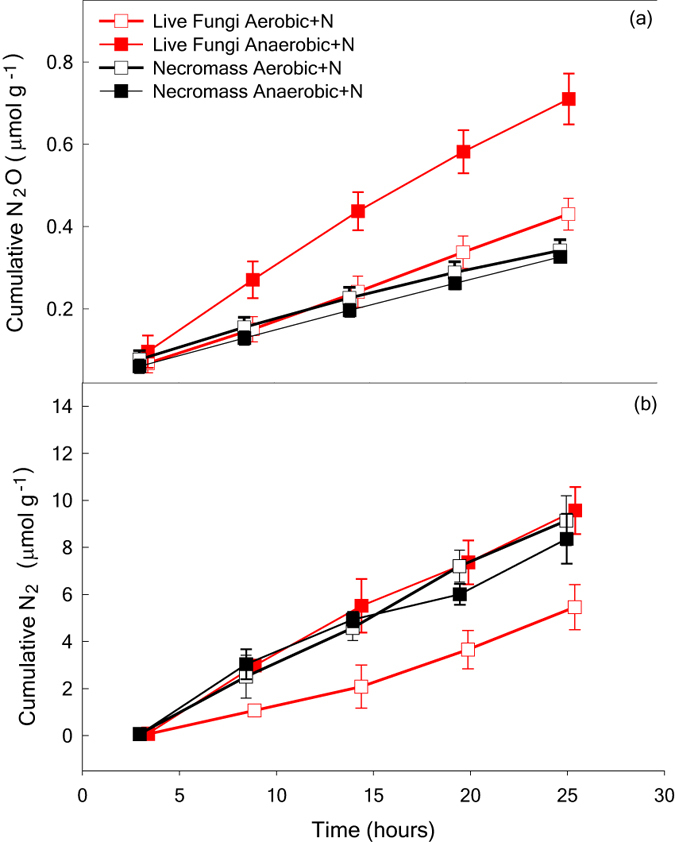
Kinetics of (**a**) N_2_O and (**b**) N_2_ production per g biomass over time following N addition at each time point as gases in the headspace accumulated. Average for each treatment are slightly staggered in time due to robotized measurement system, which is why comparisons were based on slopes over the entire incubation. Production rates of N_2_O and N_2_ were strongly affected by O_2_ status [O_2_ × fungal state (live or dead); p < 0.001] for those samples amended with N. Boxes represent average data; error bars, s.d.; n = 4.

**Figure 4:**
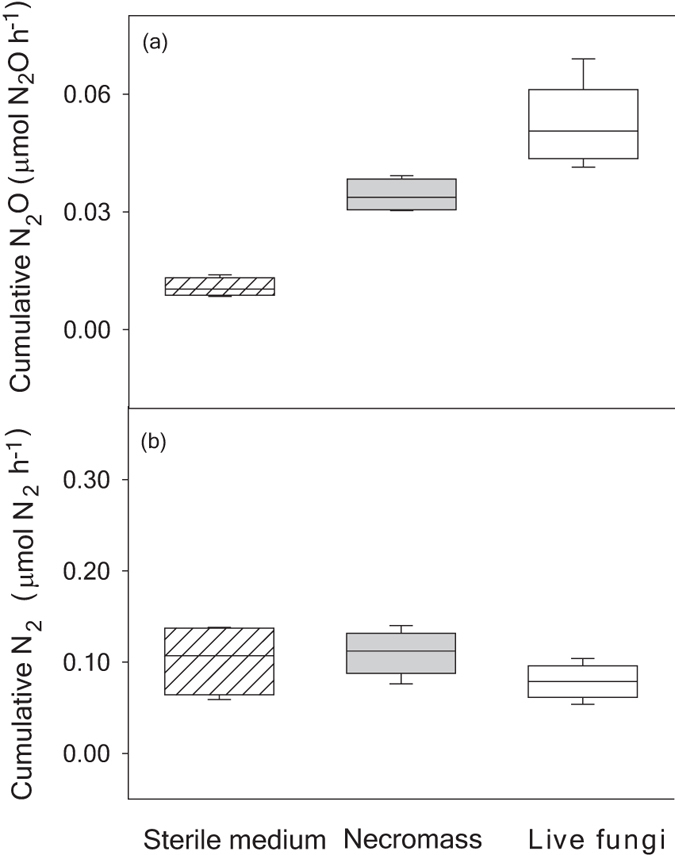
Abiotic and biotic contributions to rates of (**a**) N_2_O and (**b**) N_2_ accumulated per hour following N addition under both aerobic and anaerobic conditions for sterile medium, necromass and live fungi. Boxes represent the 90th percentile data; error bars, s.d.; n = 4. Median values are the lines horizontally bisecting each box.

Linear increases in cumulative N_2_O and N_2_ over time under aerobic and anaerobic conditions for both live fungi and necromass are shown in Fig. 3. These data suggest that N_2_O was not consumed during the incubation and both gases were produced independently. This finding contrasts with bacterial denitrification^22^, where a sharp rise in microbial production of N_2_O is followed by a rise in biological reduction of N_2_O to N_2_, and a drop in cumulative N_2_O production^21^. *Bipolaris sorokiniana* denitrifies NO_2_^−^ only and does not use glutamine to form N_2_O^16^. If the pathway to N_2_ were through the intermediate N_2_O, we would expect not only N_2_O consumption but also accumulation of ^30^N_2_ in the isotopic pairing experiment. Like anammox, our abiotic N_2_ production results indicate N_2_O formation was bypassed in the pathway to N_2_ (Fig. 1).

We show compelling evidence that formation of ^29^N_2_ does not result from solely biotic nitrosation but also abiotic nitrosation. Abiotic nitrosation occurred both in the presence and absence of O_2_, and abiotic N_2_ was formed exclusively through hybridisation of inorganic and organic N sources and did not require the N_2_O intermediary. Experimental protocols, including N concentrations, were in accordance with fungal denitrification^6^,^16^ and codenitrification^10^ studies but in the absence of soils and sediments. In soil, NO^2−^ accumulates when excess free NH_3_ inhibits bacterial NO_2_^−^ oxidation, which is often a consequence of urea hydrolysis^30^. Venterea *et al*.^34^ recovered 3–60% of added urea N as NO_2_^−^, suggesting high soil NO^2−^ is likely following urea addition (depending upon conditions and application rate). Based on these data^34^, a 1-kg bovine urine addition to soil (N concentration of 0.4 mmol kg^−1^)^35^ could result in up to 0.24 mmol NO_2_^−^. Here, we added 0.25 mmol NO_2_^−^ to 10 ml of fungal culture, which is on the high end of this scale. While we would expect abiotic formation of hybrid N_2_ via nitrosation of organic N to be more likely in grazed or fertilised agroecosystems, further N_2_ investigations with soil and at lower NO_2_^−^ levels are needed to bridge the gap between pure culture and environmental applications.

Other areas where NO_2_^−^ could accumulate include laboratory incubations where antibiotic inhibitors are added to soil and used to partition N_2_ and N_2_O production into fungal and bacterial contributions^12^,^14^. These data may be subject to artefacts if antibiotics repress NO_2_^−^ oxidation, leading to NO_2_^−^ accumulation and potential abiotic formation of N_2_ and/or N_2_O. Our report also calls into question if codenitrification alone accounts for N_2_ and N_2_O emissions following high doses of N as urea (approximately 9 mmol g^−1^ soil)^13^ and/or NO_2_^−^ (166 mmol L^−1^)^10^. If abiotic, hybrid N_2_ is formed under these conditions, our understanding of soil fungal codenitrification and N trace gas emissions would need to be re-examined. We offer new insight into chemodenitrification of NO_2_^−^ to N_2_ and present an alternative ‘N_2_O bypass pathway’. Finally, we conclude the potential for aerobic, abiotic removal of excess reactive N in terrestrial and aquatic ecosystems presents environmental mitigation research opportunities, particularly where transitory or chronic NO_2_^−^ accumulation occurs.

## Methods

Fungal incubations were conducted as described in Phillips *et al*. (2016) with the same culture ICMP 6809 isolated as a pathogen of *Hordeum distichon* in New Zealand. (https://scd.landcareresearch.co.nz/Specimen/ICMP_6809) GenBank: KU194490. We used laboratory incubations and controlled O_2_ status to evaluate if rates of N_2_O, N_2_ and CO_2_ production rates vary with aerobic conditions, as commonly reported for bacteria^20^. Experimental design was similar to previous work^16^, with 4 replicates for each treatment (N with and without O_2_; no N with or without O_2_). Cultures were grown in medium similar to other fungal denitrification studies^9^,^10^,^31^, consisting of 1% glucose, 0.2% peptone and inorganic salts^5^,^36^. The only N source in this medium used for fungal growth was peptone. One day prior to the experiment, the growth medium was washed off cultures and replaced with the same medium that was identical except it was free of peptone and so contained no N. Using the peptone-free medium herein, two amendment solutions were prepared for fungal inoculation: (a) media without N and (b) 0.25 mmol N as NaNO_2_ and 0.25 mmol N as C_5_H_10_N_2_O_3_. Nitrogen concentration was 0.04 mmol N L^−1^. Necromass was obtained by heating live *B. sorokiniana* at 60 °C for 48 h. Cell death was confirmed by (a) microscopy and (b) lack of CO_2_ respiration over a 24 h period under aerobic and anaerobic conditions. Approximately 10 ml live fungi or fungal necromass were blindly pipetted by a second independent scientist into 0.125 L serum bottles that were then amended with either media that included 0.25 mmol N as NaNO_2_ and 0.25 mmol N as C_5_H_10_N_2_O_3_ or media without N and mixed gently. Additional bottles containing sterile media solutions (with and without N) only and without fungi were also prepared. Oxygen status was controlled with airtight laboratory incubation vessels for all necromass, live fungi and media only samples. Headspace of each sample was evacuated and filled with either He (anaerobic) or heliox [aerobic (80% He, 20% O_2_)] within 2 hr following inoculation^18^,^20^. Headspace gases were quantified approximately every 6 h at 19 °C using a robotic gas chromatograph (GC) fitted with electron capture and thermal conductivity detectors according to McMillan *et al*.^21^. Helium or heliox blank standards were included in each GC run. Cumulative production rates were calculated as slopes of the masses (μmol) of N_2_O or N_2_ measured over time (h) per g of fungal biomass. Vessels incubated in He remained anoxic with the exception of necromass, where we observed 0.05% O_2_. Vessels incubated in heliox remained above 19% O_2_ with the exception of live fungi incubated without N, where 5% of the headspace O_2_ was consumed. As reported previously, addition of NO_2_^−^ inhibited O_2_ consumption and CO_2_ respiration by live fungi^16^. Fungal biomass was determined as the difference in mass with and without media after air-drying each vessel post-incubation^16^. For comparisons with sterile media, rates were also calculated as slopes of the masses (μmol) of N_2_O and N_2_ measured over time. Sterility was maintained and conditions remained constant, including pH (6.2–6.9). Sterility was tested at the end of gas sampling by pipetting 200 μL medium used in the experiment onto blood agar plates and incubating under aerobic, anaerobic (AnaeroGen^TM^, Thermo Scientific), and enriched CO_2_ (3.5–9%; CO_2_Gen^TM^, Thermo Scientific) conditions. Further, medium was also pipetted onto yeast nutrient agar, brain heart infusion agar, and potato dextrose agar plates under aerobic conditions. No evidence of fungal or bacterial growth was observed following 3, 7, and 10 d incubations at 26 °C. Data were analysed to test for effects of O_2_ and live fungi on either N_2_O or N_2_ production rates with a generalised linear model. We analysed only those samples amended with N because samples without N did not produce N_2_O or N_2_. Log transformations were employed when data did not meet the assumptions of normality. Treatments variances met assumptions of homoscedasticity. All interactions were tested and remained in the model if significant.

Di-nitrogen isotopes were evaluated in a separate experiment to assess sources of N used in abiotic production of N_2_ by adding Na^15^NO_2_ and C_5_H_10_N_2_O_3_ to sterile medium at neutral pH, as described above. We aimed to determine if N_2_ would be produced through combination of ^15^NO_2_ only (thus forming ^30^N_2_) or through combination of both C_5_H_10_N_2_O_3_ and ^15^NO_2_ (thus forming the hybrid ^29^N_2_) for N-enriched medium relative to no-N medium only. In this experiment, we used five replicates at three levels of N: (a) no-N, (b) 0.25 mmol NaNO_2_ and 0.25 mmol C_5_H_10_N_2_O_3_, and (c) 0.5 mmol NaNO_2_ and 0.5 mmol C_5_H_10_N_2_O_3_. We used the same concentration of N in each N treatment but doubled the mass of N added. Vials were prepared under aerobic and anaerobic conditions as described previously and accompanied by He blanks. The aerobic experiment was conducted separately from the anaerobic experiment, which obviated testing for effects of O_2_ on masses of ^29^N_2_ and ^30^N_2_. Headspace ^29^N_2_ and ^30^N_2_ were measured on a continuous-flow isotope ratio mass spectrometer (Thermo Finnigan Delta V, Thermo Scientific) in line with an automated gas bench interface (Thermo Gas Bench II). Precision of the isotopic analysis was <0.001atom%.

## Additional Information

**How to cite this article**: Phillips, R. L. *et al*. Chemical formation of hybrid di-nitrogen calls fungal codenitrification into question. *Sci. Rep.* **6**, 39077; doi: 10.1038/srep39077 (2016).

**Publisher's note:** Springer Nature remains neutral with regard to jurisdictional claims in published maps and institutional affiliations.

## Figures and Tables

**Figure 2:**
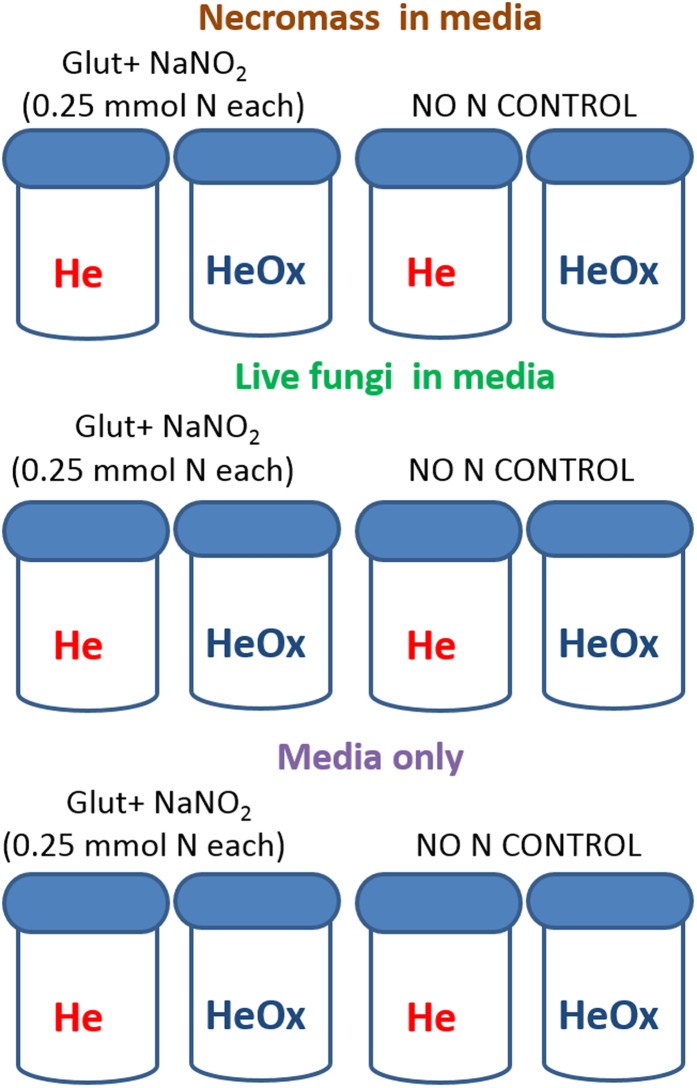
Experimental set-up to test how live fungi, necromass and media only incubated in an anoxic and 20% O_2_ atmosphere affects production of N_2_O, CO_2_, O_2_, and N_2_ following addition of both organic and inorganic forms of N to pure cultures under aseptic conditions. Terms are defined as: Glut, glutamine; NaNO_2_, sodium nitrite; He, helium; HeOx heliox.

**Table 1:** Average (SD) of ^29^N_2_ and ^30^N_2_ recovered in the headspace following aerobic and anaerobic incubation of sterile media at three levels of N addition, where added N comprised 50% N from unlabelled C_5_H_10_N_2_O_3_ and 50% N from labelled ^15^N-NaNO_2_ (n = 5).

N addition(mmol N)	^29^N_2_ (^14^N,^15^N)>(μmol) aerobic	^30^N_2_ (^15^N,^15^N)(μmol) aerobic	^29^N_2_ (^14^N,^15^N)(μmol) anaerobic	^30^N_2_ (^15^N,^15^N)(μmol) anaerobic
0	−0.012 (<0.001)	0.000 (0.000)	−0.005 (0.005)	<0.001 (<0.001)
0.5	16.274 (2.045)	0.001 (<0.001)	15.759 (0.995)	0.003 (0.002)
1.0	40.289 (2.318)	0.002 (<0.001)	31.025 (3.144)	0.007 (0.001)

Samples were incubated for >7 d prior to isotopic analyses. Values were adjusted according to helium or heliox blanks.
