# Corrigendum: Chemical formation of hybrid di-nitrogen calls fungal codenitrification into question

**DOI:** 10.1038/srep46908

**Published:** 2017-12-22

**Authors:** Rebecca L. Phillips, Bongkeun Song, Andrew M. S. McMillan, Gwen Grelet, Bevan S. Weir, Thilak Palmada, Craig Tobias

Scientific Reports 6: Article number: 39077; 10.1038/srep39077 published online: 12 15 2016; updated: 12 22 2017.

This Article contains typographical errors in the Introduction section.

“We performed a second incubation (illustration of this incubation design not shown) using isotope pairing techniques to determine if ^29^N_2_O or ^30^N_2_O were produced abiotically from sterile medium amended with glutamine and NO_2_^−^ in the presence or absence of O_2_”.

should read:

“We performed a second incubation (illustration of this incubation design not shown) using isotope pairing techniques to determine if ^29^N_2_ or ^30^N_2_ were produced abiotically from sterile medium amended with glutamine and NO_2_^−^ in the presence or absence of O_2_”.

